# Cadmium (Third Edition) (Chemicals and Contaminants)

**DOI:** 10.14252/foodsafetyfscj.D-24-00011

**Published:** 2024-09-27

**Authors:** Food Safety Commission of Japan

## Abstract

Exposure to cadmium tends to be higher in Japan than in other countries due to the wide spreading of ore deposits and many mines throughout Japan. The proximal tubule of the kidney is recognized as the most susceptible site to be affected by cadmium exposure. Food Safety Commission of Japan (FSCJ) considered it appropriate to investigate studies of the effects of cadmium intake on renal proximal tubular function using urinary β_2_-microglobulin concentration of 1,000 μg/g creatinine as a criterion for the dysfunction. Based on these epidemiological studies, FSCJ established a tolerable weekly intake (TWI) of 7 µg/kg bw per week for cadmium. This value is derived from the results of actual dietary surveys and urinary β_2_-microglobulin concentrations in Japan, and not from the results of application of blood or urinary cadmium levels to theoretical models. Therefore, no uncertainty factor is required. The estimated dietary intake of cadmium in 2022 was 2.03 µg/kg bw per week, approximately 30% of the TWI of 7 µg/kg bw per week. Therefore, it is unlikely that dietary cadmium intake in the general Japanese population would cause adverse health effects.

## Conclusion in Brief

Cadmium occurs widely in the natural environment, including soil, water, and air. Cadmium accumulates in various levels in foods such as cereal grains, vegetables, and marine products. Exposure to cadmium tends to be higher in Japan than in other countries due to the wide spreading of ore deposits and many mines throughout Japan.

The dietary intake of cadmium among the general population in Japan was estimated to be 46 μg/person per day in the late 70s and then decreasing as estimated by National Institute of Health and Science (NIHS)-based market basket studies^*1^. Currently available data from the market basket and duplicate diet studies indicate that the dietary intake of cadmium in Japan after 2010 was estimated to be 13–16 μg/person per day (0.23–0.29 μg/kg bw per day or 1.6–2.0 μg/kg bw per week as divided by the mean body weight of 55.1 kg). “Rice and Rice products” (34.1%) was the most prominent contributor to total cadmium intake in the 2022 survey, followed by “Other Vegetables, Mushrooms and Seaweed” (20.5%) (Table 1).^2^

**Table 1.** Food Category used for the NIHS-based market basket studies and contribution to total cadmium intake in the 2022

Group	Food Category
1	Rice and Rice products
2	Cereals, Seeds and Potatoes
3	Sugars and Confectioneries
4	Fats and Oils
5	Pulses
6	Fruits
7	Green Vegetables
8	Other Vegetables^#^, Mushrooms and Seaweeds
9	Beverages
10	Fish and Shellfish
11	Meat and Eggs
12	Milk and Dairy products
13	Other Foods (e.s. Seasonings)
14	Drinking water

# Excluding Colored Vegetables such as Carrots, Pumpkins, Spinach, Tomatoes, Green peppers, etc.

The proximal tubule of the kidney is recognized as the most susceptible site to be affected by cadmium exposure. Food Safety Commission of Japan (FSCJ) considered it appropriate to investigate the effects of cadmium intake on renal proximal tubular function.

For the calculation of the tolerable intake of cadmium, many risk assessment organizations used theoretical models to predict cadmium intake from urinary cadmium concentrations. Urinary cadmium concentration is affected by cadmium accumulation in the kidneys but also by other factors such as age. In addition, once proximal tubular dysfunction occurs, cadmium excretion into urine increases due to impaired reabsorption. The relationship between urinary cadmium concentration and cadmium intake is intricate, and thus the cadmium intake calculated using theoretical models such as the one-compartment model may not be highly reliable.

In Japan, epidemiological studies have been done on the association between dietary cadmium intake and the prevalence of proximal tubular dysfunction in residents of cadmium-polluted and non-polluted areas. Based on such epidemiological studies, FSCJ established a tolerable weekly intake (TWI) of 7 µg/kg bw per week for cadmium using urinary β_2_-microglobulin concentration of 1,000 μg/g creatinine as a criterion for the dysfunction. This value is derived from the results of actual dietary surveys and urinary β_2_-microglobulin concentrations in Japan, and not from the results of application of blood or urinary cadmium levels to theoretical models. Therefore, no uncertainty factor is required.

In the third edition, we also discussed epidemiological studies conducted in Sweden. These studies showed decreased bone mineral density and increased incidence of bone fractures at urinary cadmium concentrations of 0.5 µg/g creatinine or higher. Nordic countries, including Sweden, are known to have the highest age-adjusted incidence of proximal femoral fracture in the world, according to an international comparative study. Japan, however, had lower fracture incidences than those of Nordic countries. Urinary cadmium concentrations in the Japanese population in non-polluted areas before 2010 were approximately 2.0 µg/g creatinine. During that period, Sweden had the highest incidences of proximal femoral fractures in each age group among European countries, while the incidences in Japan were comparable to those in the lowest-level countries in Europe.

A recent report from an overseas risk assessment organization focused on decreased bone mineral density and increased incidence of bone fractures as endpoints in the evaluation of cadmium based on these Swedish epidemiological studies. To evaluate the bone effects of low-level cadmium exposure in the Japanese population, FSCJ considered data from Japan were essential. However, no studies were available to assess the effects of low-level cadmium exposure on bone mineral density and fracture incidence in Japan. Neither bone mineral density nor fracture incidence was, thus, used as the endpoint for cadmium exposure in this evaluation.

The estimated dietary intake of cadmium in 2022 was 2.03 µg/kg bw per week, approximately 30% of the TWI of 7 µg/kg bw per week. Therefore, it is unlikely that dietary cadmium intake in the general Japanese population would cause adverse health effects.
